# The Novel Antitubulin Agent TR-764 Strongly Reduces Tumor Vasculature and Inhibits HIF-1α Activation

**DOI:** 10.1038/srep27886

**Published:** 2016-06-13

**Authors:** Elena Porcù, Luca Persano, Roberto Ronca, Stefania Mitola, Roberta Bortolozzi, Romeo Romagnoli, Paola Oliva, Giuseppe Basso, Giampietro Viola

**Affiliations:** 1 Dipartimento di Salute della Donna e del Bambino, Laboratorio di Oncoematologia pediatrica, Università di Padova, 35128 Padova, Italy; 2 Dipartimento di Medicina molecolare e traslazionale Unità di oncologia sperimentale ed immunologia. Università di Brescia, 25123 Brescia, Italy; 3 Dipartimento di Scienze Chimiche e Farmaceutiche, Università di Ferrara, 44121 Ferrara, Italy

## Abstract

Tubulin binding agents (TBAs) are commonly used in cancer therapy as antimitotics. It has been described that TBAs, like combretastatin A-4 (CA-4), present also antivascular activity and among its derivatives we identified TR-764 as a new inhibitor of tubulin polymerization, based on the 2-(alkoxycarbonyl)-3-(3′,4′,5′-trimethoxyanilino)benzo[*b*]thiophene molecular skeleton. The antiangiogenic activity of TR-764 (1–10 nM) was tested *in vitro* on human umbilical endothelial cells (HUVECs), and *in vivo*, on the chick embryo chorioallantoic membrane (CAM) and two murine tumor models. TR-764 binding to tubulin triggers cytoskeleton rearrangement without affecting cell cycle and viability. It leads to capillary tube disruption, increased cell permeability, and cell motility reduction. Moreover it disrupts adherens junctions and focal adhesions, through mechanisms involving VE-cadherin/β-catenin and FAK/Src. Importantly, TR-764 is active in hypoxic conditions significantly reducing HIF-1α. *In vivo* TR-764 (1–100 pmol/egg) remarkably blocks the bFGF proangiogenic activity on CAM and shows a stronger reduction of tumor mass and microvascular density both in murine syngeneic and xenograft tumor models, compared to the lead compound CA-4P. Altogether, our results indicate that TR-764 is a novel TBA with strong potential as both antivascular and antitumor molecule that could improve the common anticancer therapies, by overcoming hypoxia-induced resistance mechanisms.

Microenvironment is very important for tumor sustenance, and angiogenesis is essential for tumor growth and spreading. The characterization of this process has been fundamental to design and optimize new therapies targeting both the tumor mass and the tumor blood supply, as supported by recent advanced in chemotherapy, focused on combination treatments^1^,^2^,^3^.

Tumor vasculature presents abnormal and disorganized structures, lacking of the conventional blood vessel hierarchy. Arteries, capillaries and venules are not recognizable, and they are tortuous, hyperpermeable and immature^4^. Therefore the antiangiogenic therapy targeting the vascular endothelium results very efficacious and selective.

Numerous mechanisms regulate tumor angiogenesis, and a series of molecular mediators are involved in this process, including signal transduction systems mediated by growth factors, proteins for cytoskeleton remodeling, paracrine and intracellular signaling pathways^5^.

Hypoxia inducible factors (HIFs), the key molecules regulating hypoxic stimuli appear in their active form under low oxygen tension conditions. HIFs consist of multiple isoforms, among which HIF-1α and HIF-2^6^. It has been described that hypoxic microenvironment gives rise to proangiogenic factors requirement, which in turn recruit endothelial cells and stimulate sprouting and development of new tumor blood vessels. Moreover, hypoxia is responsible for some described resistance mechanisms, which make conventional therapies ineffective^2^,^7^.

Several antiangiogenic strategies have been studied, most of them target the VEGF signaling system and are directed to tyrosine kinase receptors (i.e. VEGFR, and PDGFR)^2^, inhibiting the proliferation of new blood vessels. Other therapies target pre-existing tumor vasculature, and are principally represented by the so-called vascular disrupting agents (VDAs)^1^.

Among this class of compounds, tubulin binding agents (TBAs) represent an important group of drugs commonly used in cancer therapy as antimitotics, since recently it has been described that combretastatin A4 (CA-4) exhibits in addition to its antimitotic properties also important antivascular activity^8^.

TR-764 is a new inhibitor of tubulin polymerization, based on the 2-(alkoxycarbonyl)-3-(3′,4′,5′-trimethoxyanilino) benzo[*b*]thiophene molecular skeleton (Supplementary Fig. S1). It was selected among a series of benzothiophenes derivatives for its high antiproliferative activity *in vitro*, being endowed with GI_50_ values in the nanomolar range in different cancer cell lines^9^. Moreover it has demonstrated its ability to significantly inhibit *in vivo* the growth of a syngeneic hepatocellular carcinoma in Balb/c mice^10^.

Here we investigated the antiangiogenic activity of TR-764 in HUVEC cells, and its strong effect *in vivo* as a vascular disrupting agent, in the chick chorioallantoic membrane (CAM) and in a murine and human model of melanoma.

This compound is proposed for deepen its activity as single agent in clinical trials, with a dual effect against cancer cells as an antimitotic, and targeting the tumor vasculature exploiting its antiangiogenic activity. Traditional chemotherapy could be improved without falling back upon combination treatments, and overcoming possible resistance mechanisms.

## Results

### TR-764 has a strong antivascular effect *in vitro*

**Figure 1:**
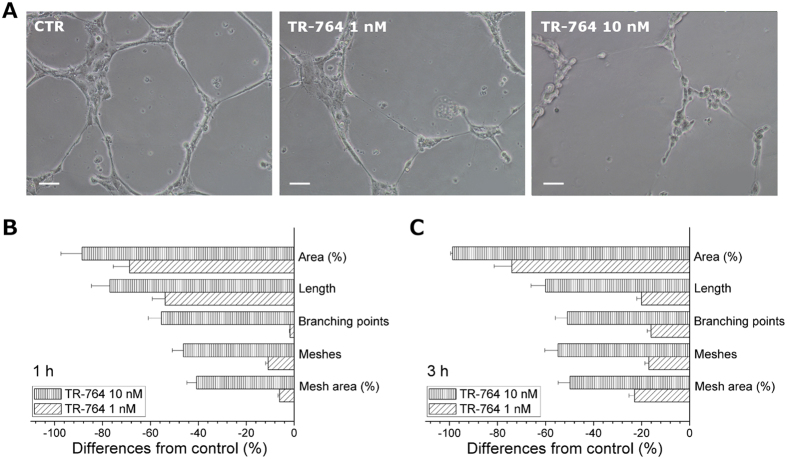
TR-764 disrupts tubule-like structures formed by HUVECs seeded on Matrigel matrix. Representative pictures of HUVECs treated with TR-764 at the indicated concentrations for 1 h (10× magnification, scale bar = 100 μm) (**A**). Quantitative analysis of TR-764 treatment for 1 h (**B**) or 3 h at the indicated concentration (**C**) on dimensional and topological parameters of tubule networks. Data were represented as mean ± SEM of three independent experiments.

HUVEC cells were used as a model to study the angiogenic process *in vitro*, since they organize themselves in tubule-like structures when seeded on a Matrigel matrix. In the presence of TR-764, at very low concentrations (1–10 nM), we observed a rapid disruption of the tubes network within 1 h of treatment, as shown in Fig. 1A, that increased after 3 h. Quantitative image analysis showed that the compound remarkably decreased both dimensional (percent of area covered by HUVECs, total length per field) and topological parameters (number of meshes per field, and number of branching points) of the capillary-like network (Fig. 1B,C).

### Endothelial cells motility is impaired by TR-764

**Figure 2:**
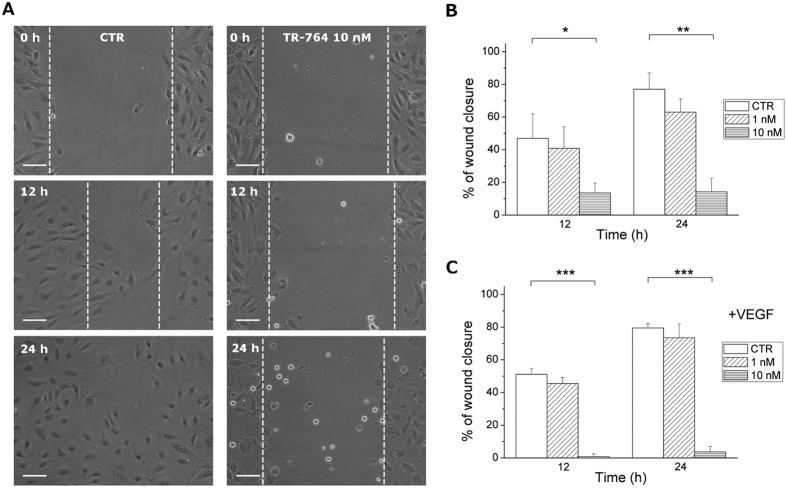
TR-764 impairs HUVECs motility in the absence and in the presence of VEGF. Confluent HUVECs monolayer was scratched and treated with TR-764 at the indicated concentrations. At different time points cells were photographed (representative images of cells without VEGF, 10× magnification, scale bar = 100 μm) (**A**) and quantified as described in the experimental section. Quantitative analyses of the effects on wound closure after treatment with TR-764 alone (**B**) or in presence of VEGF (**C**). Cell motility was quantified by measuring the scratch gap at the set times. *p < 0.05, **p < 0.01, ***p < 0.001 *vs* control.

Cell motility is a fundamental mechanism involved in angiogenesis and it was assessed by a wound healing assay, in which HUVECs monolayer was scratched and cells were allowed to migrate and restore the monolayer. As shown in Fig. 2(A,B), TR-764 at the concentration of 10 nM significantly arrested the movement of cells preventing the closure of endothelial monolayer while at the lowest concentration used (1 nM) the effect is not significant. Co-treatment with VEGF, that induces further stimuli for endothelial cell migration, did not prevent TR-764 activity, and the effect on cell motility resulted even more statistically significant at 10 nM (Fig. 2C).

### TR-764 does not alter HUVEC cell cycle and cell viability

As previously described^10^, a competitive binding cell-free assay revealed that TR-764 binds tubulin in the same binding site of colchicine, with a very strong affinity constant comparable to that of reference compound CA-4.

α-tubulin staining in HUVEC cells with a specific antibody for immunofluorescence analysis showed that tubulin filaments were completely disassembled while the cell nuclei were intact after 6 h of treatment with TR-764 (Supplementary Fig. S2), at concentration (10 nM) that, as described above, inhibited microvessels formation on matrigel and reduced cell migration.

Although TR-764 binds to the microtubules, it did not affect cell cycle and cell viability of HUVEC cells at the same concentration that impaired endothelial cells functions. As shown in Supplementary Fig. S3, no perturbation of the cell cycle, evaluated by PI staining, were identified after 24 h of treatment (Fig. S3A). Secondly, cell viability, measured using MTT dye, was only slightly impaired after 72 h of incubation (Fig. S3B). It is interesting to note that cell proliferation was only 30% reduced, at the highest concentration used (100 μM). Altogether these results indicate that, although TR-764 rapidly impairs cell cytoskeleton, it exerts antivascular effects without impairing endothelial cells survival or proliferation at the set doses and times.

### Actin cytoskeleton is rearranged by TR-764 treatment

**Figure 3:**
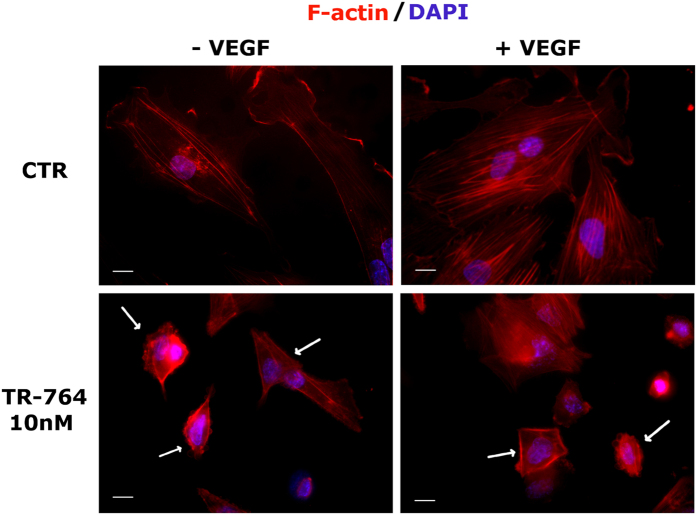
TR-764 induces cytoskeleton rearrangement. HUVECs monolayer was scratched and treated with TR-764 in the absence or in the presence of VEGF. After 6 h cells were fixed and stained with phalloidin-TRITC conjugate to visualize F-actin (red). DAPI was used to visualize cell nuclei (60× magnification, scale bar = 10 μm). In control cells lamellipodia are visible, while in treated cells blebbing occurs, as well as actin stress fibers are induced by TR-764 treatment (indicated by the arrows).

TR-764, targeting specifically tubulin, was able to disrupt microtubules, but it also induced microfilaments rearrangement. To evaluate the effects of the drug on the organization of the cytoskeleton of endothelial cells, HUVECs monolayer was scratched and cells were allowed, in the absence or in the presence of VEGF, to move into the wound. As reported in Fig. 3, after 6 h of treatment with the compound, at a concentration that impairs cell migration, endothelial cells showed a remarkable reorganization of actin filaments, marked by phalloidin in red. TR-764 increased the number of stress fibers and it caused blebbing, a characteristic phenomenon of cytoskeleton uncoupling to the extracellular matrix. In addition, control cells were able to move and they formed lamellipodia, that are projections of actin cytoskeleton on the leading edge of cell migration. Treated cells did not show these structures, indicating that all processes linked to cytoskeleton functionality could be compromised by the treatment also in the presence of VEGF.

### Cell spreading is reduced with a mechanism that involves FAK/Src and RhoA/Rock1 pathways

Focal adhesions are necessary for cell adhesion and cell migration and in particular to regulate endothelial cell spreading. One important protein is FAK, a 125-kDa cytoplasmic tyrosine kinase that is localized to focal adhesions where it acts to integrate growth factors and integrin signals^11^. FAK becomes phosphorylated after the engagement of integrins with the proteins of the extracellular matrix (ECM) and these phosphorylations are important for cell migration and invasiveness^12^,^13^.

**Figure 4:**
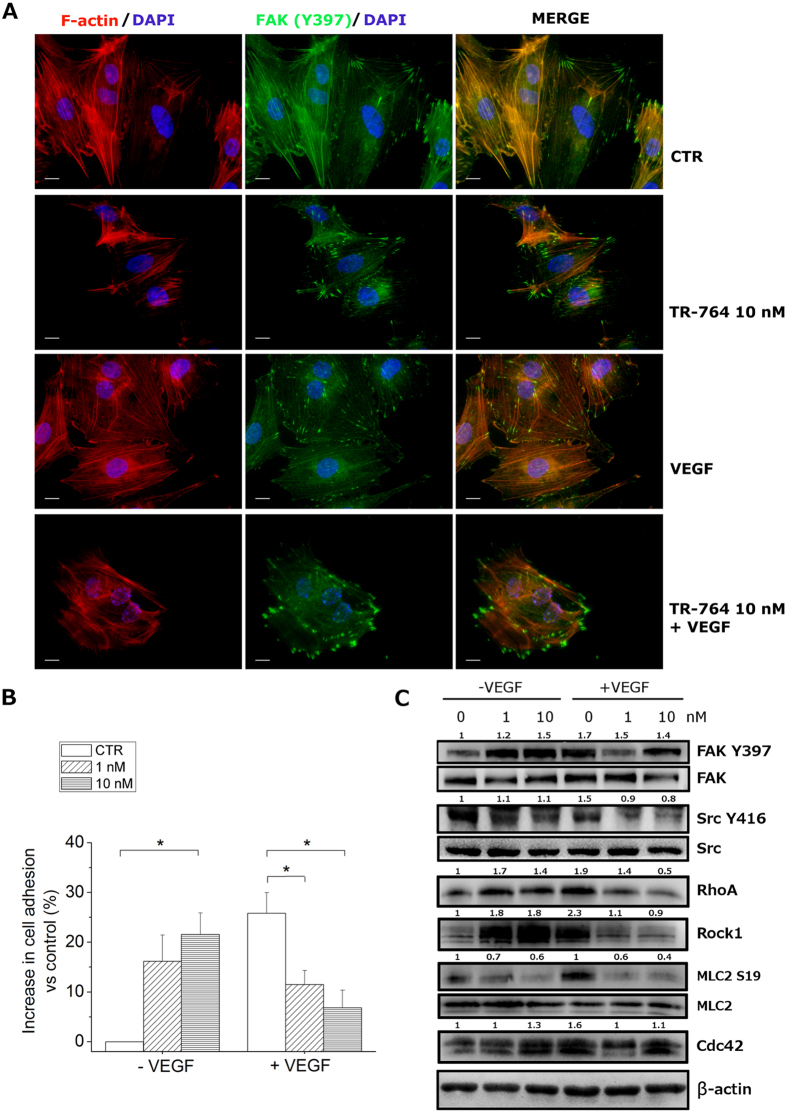
TR-764 alters cell adhesion impairing focal adhesions. HUVECs were fixed after 6 h of treatment with TR-764, and stained with phalloidin-TRITC conjugate to mark F-actin (red) and with primary antibody anti- p-FAK Y397 (green). DAPI was used to visualize cell nuclei (60× magnification, scale bar = 10 μm). In control cells and after VEGF treatment focal adhesions are well structured and strictly in contact with actin filaments. They are altered by TR-764 treatment, and they assume a more enlarged morphology (**A**). Cells were treated with TR-764 both in the absence and in the presence of VEGF for 6 h, removed from the plate and left to adhere for 30 min. Data represent mean ± SEM of three independent experiments. *p < 0.05 *vs* control (**B**). TR-764 effects on indicated proteins, after 6 h of treatment. Protein lysates were analyzed by western blot and β-actin was used as reference protein for equal loading. Densitometric analyses are reported above each lane. Phospho-proteins were normalized on the corresponding total proteins; total proteins were normalized on β-actin (**C**).

Since TR-764 is able to reduce endothelial cell migration we examined its effects on FAK disposition inside the cells and its phosphorylation status after treatment. Focal adhesions are hard-wired to actin microfilaments, as shown in the control cells in Fig. 4A. VEGF did not impair these connections, but after treatment with TR-764 the normal conformation of focal adhesions is modified, and they seem to lose the direct contact with actin, assuming a more enlarged shape. Given this effect, to better underlie the functional activity of the drug, we investigated the capability of HUVEC cells to adhere to the substrate. TR-764 alone, as reported for other VDAs^14^, was able to induce an increase in cell adhesion (Fig. 4B). On the contrary, the co-treatment with the drug and VEGF, which significantly increases cell adhesion, restored the normal amount of adherent cells (Fig. 4B). Thus, focal adhesion expanded shape induced by TR-764 can be considered as a dysfunctional form of adhesions to the extracellular matrix, that could contributed to cell spreading reduction induced by the drug.

TR-764 exerts this effect by regulating several proteins belonging to the FAK/Src axis. Figure 4C showed that, without pro-angiogenic stimuli, the treatment with TR-764 induced the activation of FAK by increasing its phosphorylated form in Y397, in a concentration dependent manner after 6 h of treatment. Moreover, a lot of downstream proteins resulted upregulated, suggesting as TR-764 activated a signaling cascade starting from the cytoskeleton organization, the crosstalk between integrins, and several proteins implicated in cell motility, adhesion and migration, such as Src, RhoA, Rock1, MLC2 and Cdc42.

Proangiogenic factors, such as VEGF, are described as inducer of cell motility, and activators of these molecules. TR-764 treatment, even at 1 nM, exerted its activity also in presence of VEGF.

In these conditions, as though FAK, the steroid receptor coactivator (Src), which regulates the focal adhesions, is dephosphorylated in Y416 by the treatment with TR-764, at the concentration of 10 nM, while total levels of FAK and Src were not significantly modified. It has been reported that phosphorylated FAK/Src interplay with the Rho protein family. RhoA is a small guanosine triphosphate hydrolase (GTPase) belonging to the Ras homology (Rho) family. Among its function, RhoA promotes contractile actin and myosin stress fibers formation and focal adhesion assembly, by the Rho associated kinase Rock1. As depicted in Fig. 4C, in the presence of VEGF, the expression of both RhoA and Rock1 were decreased by the treatment with TR-764, which strongly counteracts the pro-angiogenic stimuli. Rock1 also phosphorylates Myosin Light Chain 2 (MLC2) in S19, regulating the assembly of stress fibers^15^. Over again, TR-764 impaired the phosphorylation status of MLC2 by about 50% also at the lowest concentration (1 nM) used.

Finally, FAK/Src signal transduction culminate to another Rho GTPase, Cdc42, regulating the formation of filopodia^16^. Figure 4C showed as Cdc42 is deregulated by the co-treatment of TR-764 and VEGF.

### TR-764 strongly increases cell permeability, inhibiting VE-cadherin/β-catenin functionality and disrupting adherens junctions

**Figure 5:**
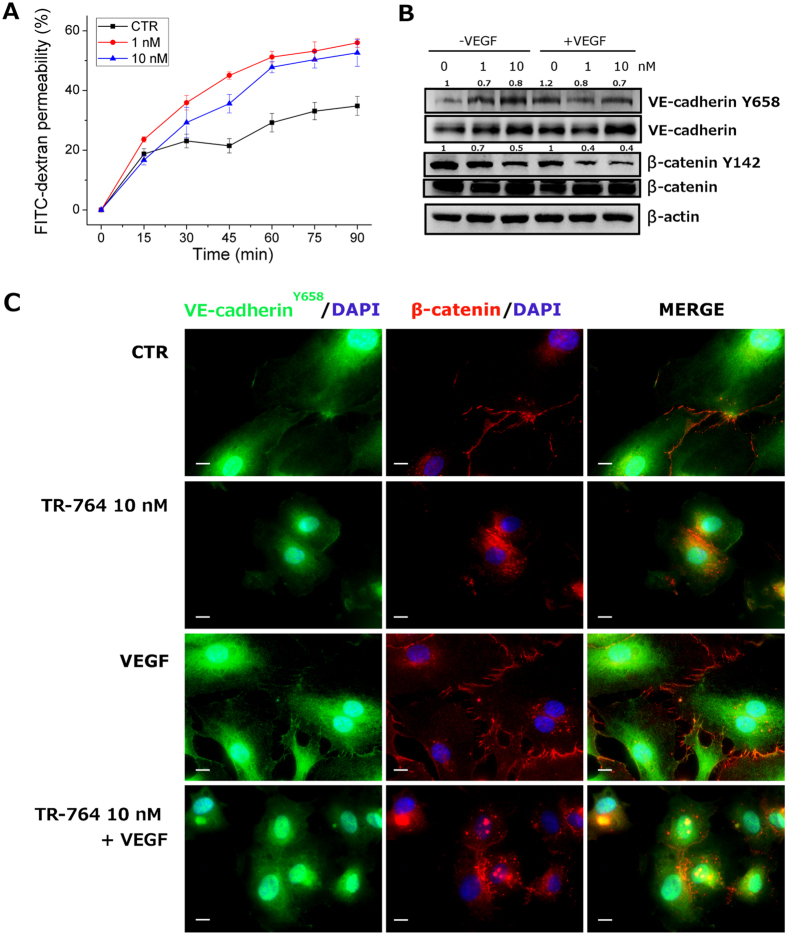
TR-764 increases endothelial cells permeability. HUVEC cells were seeded onto 24-well insert wells until a confluent monolayer was formed. They were then treated with TR-764 at the indicated concentrations. Simultaneously FITC-dextran was added and its diffusion into the lower chamber was monitored at the indicated times. Data were represented as mean ± SEM of three independent experiments (**A**). TR-764 effects on the indicated proteins, after 6 h of treatment were analyzed by western blot. β-actin was used as reference protein for equal loading. Densitometric analyses are reported above each lane. Phospho-proteins were normalized on the corresponding total proteins; total proteins were normalized on β-actin (**B**). Immunofluorescence images of HUVECs treated with TR-764 10 nM for 6 h in presence and absence of VEGF. Cells were fixed and stained with primary antibodies anti- p-VE-cadherin Y658 (green) and β-catenin (red), and DAPI was used to visualize cell nuclei (60× magnification, scale bar = 10 μm) (**C**).

The permeability of a HUVEC cells monolayer was assayed monitoring the ability of a macromolecule, dextran-FITC, to pass through the intercellular spaces within 90 min (Fig. 5A). With the increasing of time, in treated cells the permeability to dextran-FITC increased progressively, in comparison to the control.

VE-cadherin constitutes the adherens junctions and is the major endothelial cell junctional molecule mediating cell-cell interaction^17^. In addition it regulates angiogenesis, and vascular permeability, and it is also required for differentiation into vasculature-like structures by endothelial cells *in vitro.*

Since VE-cadherin is crucial for controlling the state of adherence junctions, which in turn regulate endothelial cell-cell adhesion, cell motility, morphogenesis and intracellular signaling pathways, the observed increase in cell permeability could be explained by the reduction of phosphorylation of VE-cadherin. Indeed as reported in Fig. 5B, TR-764 reduced VE-cadherin phosphorylation, both in the absence and in the presence of VEGF.

Adherens junctions are arranged not only by VE-cadherin, but also by β-catenin which also controls vascular permeability. β-catenin is directly phosphorylated by FAK in Y142, to simplify the dissociation between VE-cadherin and β-catenin and disrupt the adherens junctions^18^. TR-764 treatment gave rise to a 60% reduced β-catenin Y142 phosphorylation, maintaining the same level of the total β-catenin, as demonstrated by western blot analysis (Fig. 5B), even in the presence of VEGF. In well agreement, immunofluorescence analysis showed that both VE-cadherin and β-catenin patterns were altered. The proteins were relocalized, from ordered structures mainly linked to the adherens junctions to irregular dispositions inside the cells (Fig. 5C).

### HIF-1α activation under hypoxic conditions is impaired by TR-764 and its activity on HUVEC is preserved

**Figure 6:**
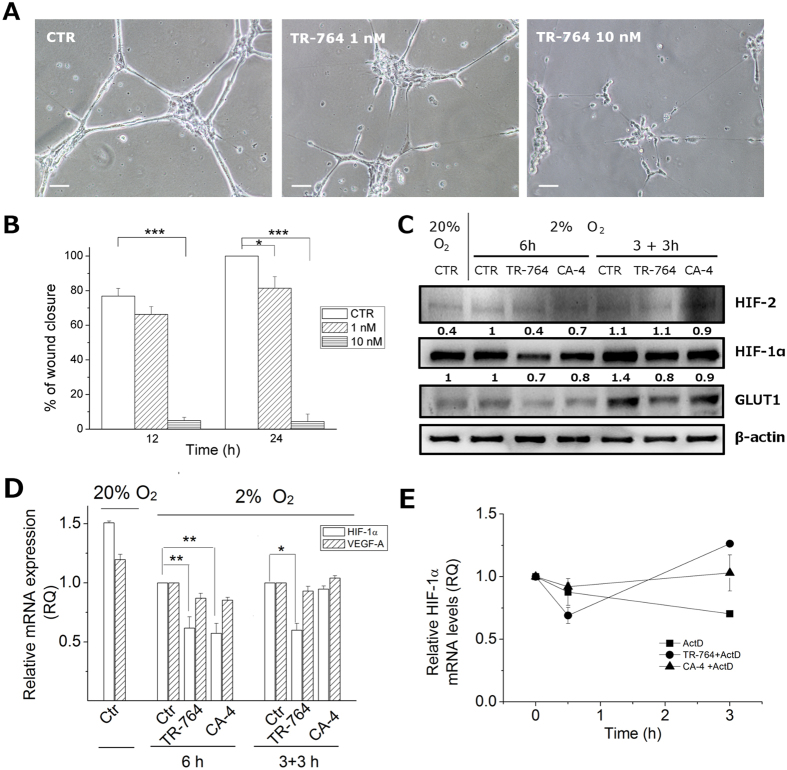
Effects of TR-764 on HUVECs in hypoxic conditions. TR-764 disrupts tubule-like structures formed by HUVECs seeded on Matrigel matrix, in hypoxia. Representative pictures of HUVECs treated with TR-764 at the indicated concentrations for 1 h (10× magnification, scale bar = 100 μm) (**A**). Confluent HUVECs monolayer in hypoxic conditions was scratched and cells were treated with TR-764, which impairs cell motility. The ability to move into the wound was measured at the indicated times, and data represent mean ± SEM of three independent experiments (**B**). TR-764 and CA-4 inhibit HIF-1α activation. Western blot analysis was performed on protein lysates of HUVECs maintained in normoxic (20% O_2_) and hypoxic (2% O_2_) conditions. On the left side of the image, treatment was simultaneously performed when HUVECs were placed in hypoxia. On the right side HUVECs were maintained in hypoxia for 3 h and subsequently treated with TR-764 and CA-4 both at 10 nM. Densitometric analyses are reported above each lane. Quantifiable data were normalized on β-actin densitometry (**C**). TR-764 inhibits HIF-1α expression, without significantly impairing VEGF-A. Relative mRNA expression was quantified by Real Time PCR and normalized on the corresponding controls, as indicated (**D**). HUVEC cells were pretreated for 1 h with the transcriptional inhibitor Actinomycin D (5 μg/ml) and then with TR-764 and CA-4 at 10 nM, for the indicated times (**E**).

TR-764 activity on endothelial cells was preserved also in hypoxic conditions, where angiogenic stimuli are upregulated^19^. As shown in Fig. 6A and S4 (Supplementary information), the tubule-like structures formed by HUVEC on Matrigel, were rapidly disrupted by TR-764, similarly to the reference compound CA-4. Furthermore, hypoxia remarkably increased HUVEC migration speed, respect to normoxia (see for comparison Fig. 2), and TR-764 was significantly more active to arrest cell motility, in particular at 10 nM (Fig. 6B).

It is well known that intratumor hypoxia is associated with cellular resistance to chemotherapy, due to overactivation of HIFs. Furthermore some studies pointed out that some TBAs are endowed with the ability to reduce HIF-1α upon treatment^20^,^21^. In this context we evaluated the effects of TR-764, compared to CA-4, on the regulation of hypoxic stimuli. HUVEC cells, maintained at 2% oxygen for 3–6 h, are able to produce the stable form of HIFs, which are degraded in normoxic conditions. Interestingly, the contemporary induction of hypoxic stimuli together with the treatment with TR-764 (10 nM) for 6 h prevented HIF-1α induction, as shown in Fig. 6C. Moreover, 3 h pre-incubation of HUVECs at hypoxic conditions leaded to HIF-1α upregulation in the control, while further 3 h of treatment with TR-764 decreased the protein activation. The glucose transporter GLUT1, directly regulated by HIF-1α and associated with angiogenesis^22^, is reduced by the treatments too. Additionally, in all conditions HIF-2 is not impaired, indicating that this isoform is not involved.

To determine if the effect of down-regulation of HIF-1α by TR-764 occurs at transcriptional level, we analyze the expression of mRNA. The results depicted in Fig. 6D indicate that in hypoxic conditions the drug is able to significantly reduce the HIF-1α mRNA levels, without significantly altering the HIF-1α downstream target VEGF-A. In addition to evaluate if the compound decreases mRNA levels affecting HIF-1α stability we pretreated for 1 h HUVEC cells with the transcriptional inhibitor Actinomycin D and then with TR-764 and CA-4 respectively. The results showed that, although a certain variability has been observed in these conditions, TR-764, as well as CA-4, do not reduce HIF-1α mRNA, suggesting that the compounds potentially act at transcriptional level and do not affect the stability of the mRNA (Fig. 6E).

### FGF-induced vascularization in CAM is highly reduced by TR-764 treatment

**Figure 7:**
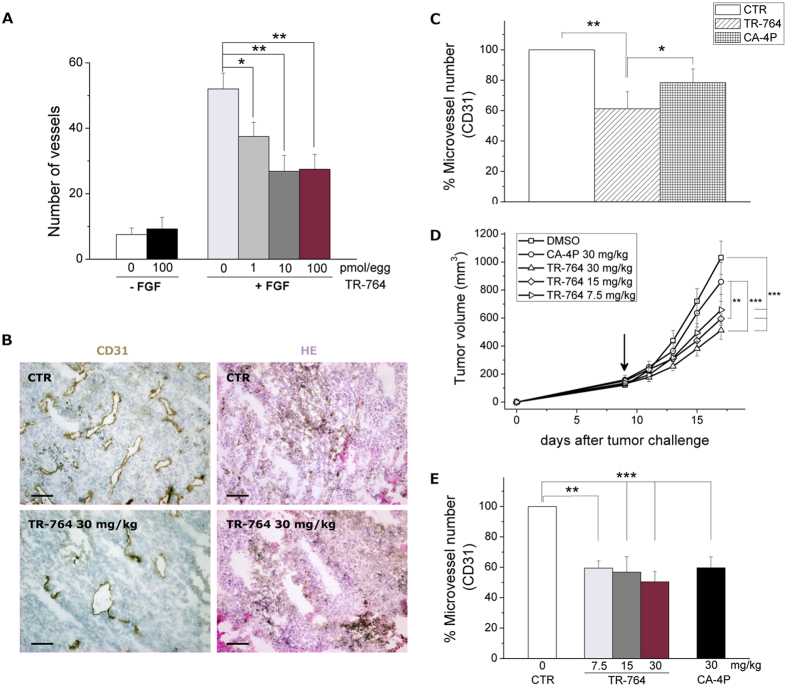
TR-764 significantly reduces the vasculature and inhibits the growth of tumor mass *in vivo*. TR-764 reduces the number of vessels on chorioallantoic membrane (CAM) assay. Alginate sponges embedded with TR-764 at the indicated doses in the absence or in the presence of FGF (100 ng) were implanted on the top of the growing CAM on day 11 of development. On day 14, newly formed blood vessels converging toward the implants are counted at microscopic levels (**A**). BL6-B16 murine melanoma cells were injected in the right flank of C57BL/6 mice. Tumor tissues were embedded in OCT-compound and frozen. CD31 immunohistochemistry and hematoxylin–eosin (HE) staining of tumor after i.p. treatment with the compounds at the indicated doses, for 24 h (10× magnification, scale bar = 100 μm) (**B**). Quantitative analysis of tumor section stained with CD31, after treatment for 24 h (**C**). Tumor-bearing mice were administered the vehicle, as control, or the indicated doses of TR-764 or CA-4P as reference compound. Injections were given intraperitoneally every other day, starting on day 8, as indicated by the arrow. The figure shows the average measured tumor volumes of the mice recorded during the treatments. Data are presented as mean ± SEM of tumor volume at each time point for 5 animals per group (**D**). Quantitative analysis of tumor section stained with CD31 after injections every other day for 10 days (**E**). *p < 0.05, **p < 0.01, ***p < 0.001 *vs* control.

Chick embryo chorioallantoic membrane (CAM) is a highly vascularized structure, usually utilized to study angiogenesis and antiangiogenic compounds *in vivo*. To this purpose, alginate beads containing TR-764 (1-10-100 pmol/egg) were applied on the CAM at day 11 post egg fertilization, in the presence or absence of bFGF (100 ng/egg). As shown in Fig. 7A, FGF exerts a strong proangiogenic effect, causing a massive production of newly formed blood vessels, which is significantly reduced by TR-764 treatment, in a dose dependent manner even at the lowest concentration tested (1 pmol/egg). Notably, TR-764 alone at the highest concentration used did not induce any effects on blood vessels.

### Tumor vasculature and tumor growth are significantly inhibited by TR-764 in syngeneic and xenograft tumor models

TR-764 was further evaluated *in vivo* in a syngeneic murine model of melanoma where BL6-B16 cells were injected s.c. in C57BL/6 mice, and in a xenograft model where A375M human melanoma cells were injected s.c. in immunodeficient NOD/SCID mice. In a first set of experiments with the syngeneic BL6-B16 model, when tumor were palpable TR-764 was administered i.p. at a single dosage of 30 mg/Kg and after 24 h tumors were excised and blood vessels were stained for the endothelial marker CD31 and counted. As depicted in Fig. 7B,C, a single injection of TR-764 is able to significantly reduce the number of vessels by 40%, and this effect was duplicated respect to that of CA-4P, used as reference compound.

To better investigate the antitumor potential of TR-764, BL6-B16 tumor bearing mice were treated, every other day i.p. with different doses (7.5, 15, and 30 mg/kg respectively) of TR-764, and compared with CA-4P (30 mg/kg). As shown in Fig. 7D after 10 days of treatment, CA-4P slightly decreased tumor growth, while TR-764 was able to significantly impair tumor burden in a dose-dependent manner and at all doses tested. Interestingly, the maximum dosage (30 mg/kg) of TR-764 reduced 50% of the tumor mass, and the minimum dose (7.5 mg/kg) shrinked the tumor by more than 30%. Notably the *in vivo* efficacy clearly points to an increased anti-tumor efficacy of the new compound compared to the reference compound CA-4P. As shown by immunohistochemistry analyses performed on tumor pellets collected from the *in vivo* study, TR-764 is endowed with a potent anti-vascular activity. At the end of the experimental procedure and treatments, the percentage of microvessels is reduced by TR-764 of about 50% both at 30 and 15 mg/Kg doses. Notably also the lower concentration of TR-764 (7.5 mg/kg) was able to decrease the microvascular density of about 40%, similarly to the treatment with CA-4P 30 mg/kg (Fig. 7E).

**Figure 8:**
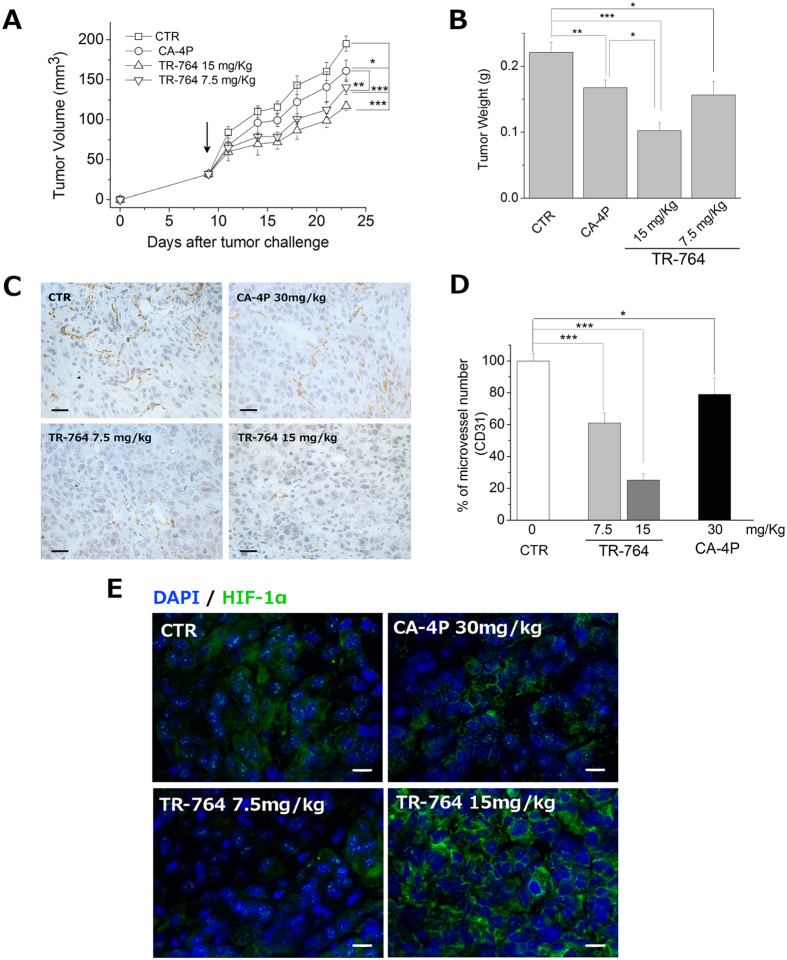
TR-764 significantly reduces the tumor growth and the vasculature *in vivo*, in a xenograft murine model. A375M human melanoma cells were injected s.c. into the dorsolateral flank of NOD/SCID mice. Tumor-bearing mice were administered with the vehicle, as control, or with the indicated doses of TR-764. CA-4P (30 mg/kg) was used as reference compound. Injections were given intraperitoneally every other day, starting on day 8, as indicated by the arrow. The figure shows the average measured tumor volumes (**A**) and tumor weight (**B**) of the mice recorded during the treatments. Data are presented as mean ± SEM of tumor volume or tumor weight for 5 animals per group (**A**,**B**). Tumor tissues were embedded in OCT-compound and frozen. CD31 immunohistochemistry staining of tumor after i.p. treatment with the compounds at the indicated doses (40× magnification, scale bar = 20 μm) (**C**). Quantitative analysis of tumor section stained with CD31, after treatment for 10 days every other day (**D**). *p < 0.05, **p < 0.01, ***p < 0.001 *vs* control. Immunofluorescence staining with primary antibody anti- HIF-1α of treated tumors for 10 days. DAPI was used to visualize cell nuclei (20× magnification, scale bar = 20 μm) (**E**).

Similar results were obtained also in the xenograft model where human melanoma (A375M) bearing mice were treated every other day with TR-764 at 15 and 7.5 mg/kg or with CA-4 P (at 30 mg/kg). As shown in Fig. 8A,B, TR-764 significantly impaired tumor growth resulting in a reduced tumor mass, at the end of the experimental procedure, of about 40% at the highest dose (15 mg/kg) and 28.2% at the dose of 7.5 mg/kg (Fig. 8A), while CA-4P at 30 mg/kg reduced the tumor volume of 18%.

In order to obtain greater accuracy in measuring the tumor masses individual tumors at the end of treatment they were removed and weighed accurately. The results presented in Fig. 8B confirm the high efficacy of TR-764 in reducing tumor burden.

The tumor vasculature was also measured by means of CD31 immunostaining and the results (Fig. 8C,D) showed a dramatic reduction of the microvessel number of about 80% and 40% at the doses of 15 and 7.5 mg/kg respectively. Although statistically significant the reference compound CA-4P induce only a modest reduction of about 22% but at higher dose (30 mg/kg). Altogether the results obtained in the two tumor model, strongly suggest that TR-764 can selectively destroy tumor vasculature. It is also important to note that even at the highest dose, TR-764 did not present any sign of toxicity and did not cause decrease of animals body weight in both tumor models (data not shown).

Since we have shown that TR-764 reduces the levels of HIF-1α in hypoxic conditions, we wanted to verify whether the drug was able to induce an impairment in the levels of HIF-1α in the tumor samples of the animals treated with the test compound. Interestingly, as shown in Fig. 8E, we can observe the presence of HIF-1α localized at the nuclear level in control tumors. On the contrary the presence of HIF-1α in the nuclei is strongly reduced by CA-4P and TR-764 especially at a dose of 15 mg/kg, where the transcription factor is shuttled to the cytoplasm.

## Discussion

Tubulin binding agents are commonly used in cancer therapy as antimitotic drugs, and recently, antivascular effects were described. Here we investigated the antiangiogenic activity of a new tubulin binding agent, TR-764, which showed a very strong activity in inhibiting the proliferation of several tumor cells *in vitro* and the tumor growth *in vivo*^10^. TR-764, binding to tubulin, triggers a series of events resulting in endothelial cells dysfunction. TR-764 revealed a marked ability to disrupt the tubule-like structures formed by HUVEC cells seeded on Matrigel, indicating its antiangiogenic activity *in vitro*. Secondly, it significantly arrested endothelial cells motility, both in presence and in absence of VEGF, as well as it caused the increase of endothelial cell monolayer permeability. Interestingly all these effects are independent from TR-764 toxicity in HUVECs, because it did not impair neither the cell cycle nor the cell viability of treated cells, at the concentration at which it exerts antiangiogenic properties, in well agreement with many reports that indicate that the inhibitory effects of TBAs on endothelial cells occur at concentration lower than that produce toxic effects^23^,^24^,^25^,^26^. Moreover the effects on microtubules occurred rapidly, within 6 h of treatment. All these alterations could potentially affect only the tumor blood vessels, composed by highly disorganized and weak structures that are more sensitive to TBAs^4^, respect to the normal vascular network, making TR-764 a selective molecule against tumor vasculature.

This new TBA efficiently disrupted microtubules structures, causing a strong rearrangement of cytoskeleton, although microfilaments remained intact. Actin filaments were also reorganized, stress fibers and blebs structures appeared after treatment. Altogether, these effects indicate an uncoupling of plasma membrane to extracellular matrix, and this action ultimately is reported to abrogate the complex system of integrating extracellular signals directed to the endothelial cells^27^.

Tubulin and actin filaments are both associated to focal adhesions, integrins and cadherins^28^,^29^. All these molecules are involved in several biological mechanisms such as cell adhesion, migration and permeability. They mediate signals deriving from the extracellular matrix or tyrosine kinase receptors, such as VEGFR2, which activate intracellular cascades, mediated by Src/FAK^30^ and culminating in the activation of the Rho family proteins RhoA and Rock1. These proteins in turn regulate myosin by phosphorylating MLC2 in S19, thus inducing the remodeling of cell contractility. Myosin chains are tightly anchored to the actin filaments and together they regulate the organization of cytoskeleton. Thus, targeting endothelial microtubules we could impair this complex signal transduction, involved in a series of processes which stimulate the vascular development.

Besides, the actin cytoskeleton is connected to the plasma membrane through VE-cadherin and one of its intracellular binding partner, β-catenin, which is another molecule critically involved in endothelial cells permeability^31^.

**Figure 9:**
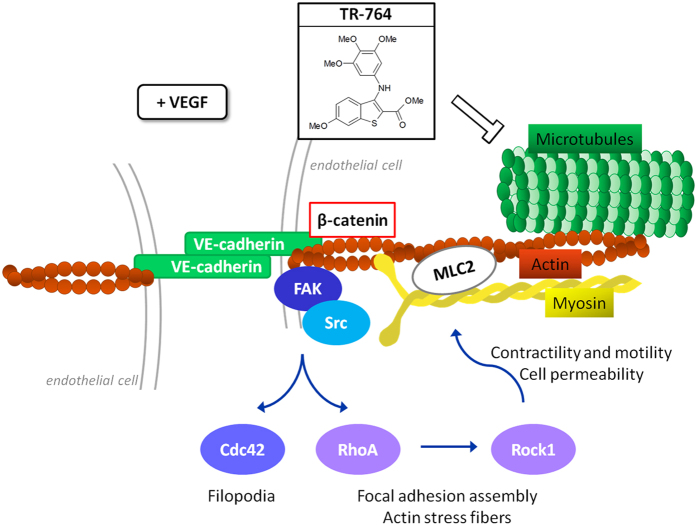
Proposed mechanism for the antivascular effects of TR-764 in endothelial cells. It directly binds to tubulin and alters the cytoskeleton organization. By impairing microtubules, TR-764 inhibits the signaling cascade activated by pro-angiogenic stimuli as VEGF, involving the adherens junctions between endothelial cells and the cellular organization of actin and myosin fibers, strictly linked to tubulin. This reorganization leads to the downregulation of several molecules such as VE-cadherin/β-catenin, MLC2, and of FAK/Src pathways, culminating in the inhibition of the Rho family proteins, giving rise to a complex alteration of mechanisms involved in cell motility, adhesion and permeability, essential for the angiogenic process.

We demonstrated that TR-764 increase cell adhesion and activation of FAK and Rho pathways, in basal conditions. On the other hand, it showed to strongly counteract pro-angiogenic stimuli, suggesting its potential role as antivascular drug. In the presence of VEGF, TR-764 gave rise to both Src and FAK inactivation, by inducing their dephosphorylation, and by triggering a signaling cascade which involves the Rho-kinases and specific molecules depicted in Fig. 9. In addition, after treatment with TR-764, β-catenin phosphorylation in Y142 was decreased, leading to its dissociation from VE-cadherin. Physiological shape of focal adhesions was impaired and adherens junctions were disrupted by TR-764, as indicated by VE-cadherin dephosphorylation in Y658. The uncoupling of VE-cadherin with β-catenin, as well as their disorganization and relocalization far from junctions, explained the elevated cell monolayer permeability induced by the treatment. Moreover it has been reported that Src family kinases stimulate VE-cadherin phosphorylation in adherens junctions, leading to increased vascular permeability^32^,^33^. Thus TR-764, starting from its binding to tubulin, induced cytoskeleton rearrangements, leading to numerous alterations in cell-to-cell contacts and in the interaction to the extracellular matrix. Accordingly, many processes involved in vascular development, such as migration and VEGF-induced stimulation, resulted impaired. A possible mechanism of action of TR-764 derived from our findings is summarized in Fig. 9.

Vessel growth, stimulated by pro-angiogenic factors, was efficiently counteracted by TR-764 also *in vivo*, as demonstrated in the CAM assay. Vascular development was massively induced by FGF and the number of vessels was strongly reduced by the treatment with TR-764 even at very low doses. Importantly, our compound alone did not show any effect on vascular growth, unlike CA-4 which is able to stimulate vessel growth in basal conditions, as we previously described^34^. TR-764 demonstrated highly significant antivascular activity also in a murine and human model of melanoma, respect to the lead compound CA-4P, both in acute and after prolonged treatment. Interestingly, TR-764 not only prevented vascular development, but also arrested the tumor growth, significantly reducing the tumor volume even at doses lower than that of reference drug in both the murine models used. In striking contrast the lead compound CA-4P, in our model, is able to reduce the tumor vasculature but not the tumor mass. This peculiarity suggests that TR-764 is a potential candidate as single agent for both anticancer and antivascular therapy. Moreover, numerous failures in recent pharmacological approaches occur, because of resistance mechanisms, mainly governed by hypoxia. Tumor microenvironment, as well as antiangiogenic therapies, give rise to hypoxic stimuli, and tumor cells adapt themselves by developing resistance^2^,^35^. Thus there is an emergent need to find new strategies to overcome tumor resistance, and in particular to target HIF-1α that is overactivated in hypoxic condition. In this context, TR-764 could be a valuable candidate, since it preserved its antiangiogenic properties *in vitro* under hypoxic conditions and it is endowed with the ability to inhibit or reduce HIF-1α activation, with the aim to prevent potential adaptive signals. TR-764, at the same concentrations that impair angiogenesis, reduced HIF-1α mRNA expression *in vitro* likely without impairing its degradation. VEGF-A belongs to the large plethora of the transcriptional targets of HIF-1α and our findings revealed that it is not significantly decreased in our conditions. A possible explanation is that it could be reduced at longer times. Moreover, VEGF-A represents a downstream target of a multiple signaling pathways, making its specific modulation a challenge. However, TR-764 showed the ability to induce the delocalization of the transcription factor HIF-1α from the nucleus to the cytoplasm in a xenograft murine model, thus triggering its inactivation and counteracting this character of tumor malignancy.

Anyway, since the goal of anticancer therapy should be to contrast the growth of tumor mass and selectively disrupt the tumor vasculature, overcoming the adaptive mechanisms, TR-764 is a potential candidate for the treatment of highly vascularized tumors.

## Methods

### Reagents and cell cultures

TR-764 was synthesized as previously described^9^. Combretastatin A-4 (CA-4) and its disodium salt phosphate prodrug (CA-4P) were synthesized as described^36^,^37^,^38^. Human VEGF (Sigma) and Actinomycin D (ActD, Sigma) were used at the final concentrations of 10 ng/ml and 5 μg/ml respectively. HUVECs were prepared from human umbilical cord veins, as previously described^39^, and maintained in M200 medium additioned by LSGS (Low Serum Growth Supplement) (Life technologies) from the first to sixth passages. They were maintained in a normoxic atmosphere of 20% oxygen, 5% carbon dioxide and balanced nitrogen, or in hypoxia (2% oxygen, 5% carbon dioxide and balanced nitrogen) as previously described^40^. BL6-B16 murine melanoma cells were maintained in DMEM supplemented with 10% fetal calf serum (FCS).

### Capillary tube formation assay

6 × 10^4^ HUVECs were incubated over Matrigel matrix (Basement membrane matrix, BD Biosciences) to allow the capillary tubes to form, as previously described^34^. Different concentrations of compounds were added in the cultures and incubated for different times and the disappearance of existing vasculature was monitored and photographed (five fields for each well: the four quadrants and the center) at a 10× magnification. Phase contrast images were recorded using a digital camera and save as TIFF files. Image analysis was carried out using the ImageJ image analysis software, as described^41^. Values were expressed as percent change from control cultures grown with complete medium.

### Wounding of endothelial cells monolayer

Motility assay for HUVECs was based on “scratch” wounding of a confluent monolayer, treated with the test compound at different times from the scratch. At all indicated time points, the cells were photographed under a light microscope (10× magnification), and the wound width was measured in four areas and compared with the initial width^42^,^43^.

### Cell cycle analysis

HUVECs cells (5 × 10^5^) were treated with different concentrations of the test compound for 24 h. After the incubation period, the cells were collected, centrifuged and fixed with ice-cold ethanol (70%). The cells were then treated with lysis buffer containing RNAse A and 0.1% Triton X-100, and then stained with propidium iodide (PI). Samples were analyzed on a Cytomic FC500 flow cytometer (Beckman Coulter). DNA histograms were analyzed using MultiCycle for Windows (Phoenix Flow Systems).

### Cell viability

Individual wells of a 96-well tissue culture microtiter plate were inoculated with 100 μL of complete medium containing 8 × 10^3^ cells, and treated with the test compounds for 72 h. Cell viability was assayed by the (3-(4,5-dimethylthiazol-2-yl)-2,5-diphenyl tetrazolium bromide test as previously described^44^.

### Colony forming assay

HUVECs were plated at 1 × 10^3^ cells/well in six-well plates to provide an optimal counting density. Cells were treated with compounds at different concentrations. After 24 h, medium was replaced with fresh one and cells were cultured for 1–2 weeks until well-defined colonies had formed (replacing culture medium every 2–3 days). Cells were briefly washed with 0.9% saline solution and stained with 0.5% crystal violet in 20% methanol. Colonies of ≥50 cells were then counted visually.

### Immunohystochemical and immunoflorescence analyses

Cells were fixed in cold 4% formaldehyde for 15 min, rinsed and stored prior to analysis. Primary antibody staining was performed for α-tubulin (Sigma-Aldrich), FAK Y397 (BD Biosciences), VE-cadherin Y658 (Abcam), β-catenin (Abcam). After incubation, cells were washed and incubated with a secondary antibody Alexa conjugated (1:2000, Life technologies). Cells were counterstained with DAPI (1:10000, Sigma-Aldrich). For F-actin visualization the cells were fixed as above and stained with phalloidin-tetramethylrhodamine B isothiocyanate conjugate (Sigma-Aldrich). Images were obtained on a video-confocal microscope (Vico, Ecliple Ti80, Nikon), equipped with a digital camera. Excised tumors were cut with a cryostat in 4–5 μm sections. Immunohistochemistry was performed by staining samples with rat anti-mouse CD31 antibody (1:200, BD Biosciences) and biotinylated goat anti-rat secondary antibody (1:100, BD Biosciences). The detection of tumor vasculature was performed using HRP-conjugated streptavidin (1:500; Jackson ImmunoResearch Laboratories). The microvessel density was evaluated by counting the number of vessels in 5 fields per section, using a 40× objective. Immunofluorescence analysis was performed with primary antibody anti- HIF-1α (1:100, Sigma), secondary antibody Alexa conjugated (1:1000, Life technologies) and DAPI (1:10000, Sigma-Aldrich). For subsequent experiments, samples were formalin-fixed and paraffin embedded and then a staining with hematoxylin and eosin (HE) was also performed to visualize the histological features of tumors. All specimens were viewed under a video-confocal microscope (Vico, Ecliple Ti80, Nikon), equipped with a digital camera, and images were captured using a 10×, 20× or 40× objective.

### Immunoblotting

Proteins were extracted by HUVECs cells, incubated in the presence of test compounds for different times. The protein concentration was determined using the BCA protein assay reagents (Pierce), equal amounts of protein (10 μg) were resolved by SDS PAGE (7.5–15% acrylamide gels) and transferred to PVDF Hybond-p membrane (GE Healthcare). Membranes were blocked with 3% BSA blocking buffer (Sigma-Aldrich) and incubated overnight at 4 °C with primary antibodies against Src Y416 (Cell Signaling), Src (Src-1M-341, Santa Cruz), FAK Y397 (Beckton Dickinson), FAK (C-20, Santa Cruz), RhoA (Santa Cruz), Rock1 (Santa Cruz), Cdc42 (Cell Signaling), β-catenin Y142 (Abcam), β-catenin (Abcam), VE-cadherin Y658 (Abcam), VE-cadherin (D87F2 XP®, Cell Signaling), MLC2 S19 (Cell Signaling), MLC2 (Cell Signaling), HIF-1α (BD Biosciences), HIF-2 (Novus Biologicals), GLUT1 (H-43, Santa Cruz), β-actin (Sigma-Aldrich). Membranes were next incubated with peroxidase-labeled secondary antibodies for 60 min. All membranes were visualized using ECL Select (GE Healthcare) and images were acquired by Alliance LD2 scan (UVITEC Cambridge). ImageJ software was used for densitometric analyses. Phospho-proteins were normalized on the corresponding total proteins; total proteins were normalized on β-actin.

### Endothelial cell adhesion

HUVECs were seeded in six-well plates to provide an optimal density and treated with compounds at different concentrations. After 24 h, cells were trypsinized and plated in quadruplicate on a 96-well plate at 5 × 10^4^ cells per well. Cells were allowed to attach for 20 min at 37 °C, and then unattached cells were gently removed. Adherent cells were washed three times with PBS and incubated with 3-(4,5-dimethylthiazol-2-yl)-2,5-diphenyl tetrazolium bromide. MTT test was performed as previously described^45^ to quantify the attached cells.

### Macromolecular permeability

HUVECs were seeded at a density of 3 × 10^5^ cells per well into 24-well cell culture inserts (1.0 μm, Falcon) and incubated for 24 h to allow a confluent cell monolayer to form. Drugs at varying concentrations were added to the cells at the upper chamber and incubated for different times at 37 °C. At the same time, FITC-dextran (Fluorescein Isothiocyanate-dextran 40 kDa, Sigma-Aldrich) was added to the upper chamber. The effects of the compound on HUVEC monolayer permeability were monitored using a fluorescent plate reader (Victor3 Perkin Elmer) as measured by increased fluorescent signal in the lower chamber as a function of time.

### Quantitative real-time RT-PCR

Relative mRNA levels were quantified by real-time RT-PCR assays, using GUS as reference gene. Total RNA was isolated using TRIzol (Invitrogen) from HUVEC cells treated with the compounds for different times. 1 μg of RNA was transcribed using the Superscript II system (Invitrogen-Gibco) according to the manufacturer’s instructions. Quantitative real-time PCR (qRT-PCR) was performed with 1 μL cDNA in 20 μL using the Sybr Green method (Applied Biosystem) and analyzed on an ABI PRISM 7900HT Sequence detection system (Applied Biosystems). The oligonucleotides to amplify mRNA fragments were HIF-1α (forward 5′–CGTTCCTTCGATCAGTTGTC–3′, reverse 3′–TCAGTGGTGGCAGTGGTAGT–5′), VEGF-A (forward 5′–AGAAAATCCCTGTGGGCCTT–3′, reverse 3′–CGTTTAACTCAAGCTGCCTCG–5′), and GUS (forward 5′–GAAAATATGTGGTTGGAGAGC–3′, reverse 3′–CGAGTGAAGATCCCCTTTTTA–5′). After normalization on GUS, expression regulation was calculated respect to untreated cells.

### Chick embryo chorioallantoic membrane (CAM) assay

Alginate pellets containing 0.1–1.0 pmol per pellet of TR-764 or CA-4 were grafted on the chorioallantoic membrane (CAM) of fertilized chicken eggs at day 11^46^. Human recombinant bFGF was purchased from Tecnogen (Piana di Monteverna, Caserta, Italy) and 100 ng were applied on alginate pellets. After 72 h new blood vessels converging toward the implant were counted at 5× magnification under a stereomicroscope.

### *In vivo* tumor model

Animal experiments were approved by our local animal ethics committee (OPBA, Organismo Preposto al Benessere degli Animali, Università degli Studi di Brescia, Italy) and were executed in accordance with national guidelines and regulations. Procedures involving animals and their care conformed with institutional guidelines that comply with national and international laws and policies (EEC Council Directive 86/609, OJ L 358, 12 December 1987) and with “ARRIVE” guidelines (Animals in Research Reporting *In Vivo* Experiments). In a first series of experiments, six week old C57BL/6 mice (Charles River, Calco) were injected s.c. into the dorsolateral flank with 2.5 × 10^5^ BL6-B16 murine melanoma cells in 200 μl-total volume of PBS. In a second one NOD/SCID mice were injected s.c. into the dorsolateral flank with 2.5 × 10^5^ A375M human melanoma cells in 200 μl-total volume of PBS. In both models, when tumors were palpable animals were treated i.p. every other day with different doses of test compounds dissolved in 100 μl of DMSO. Tumors were measured in two dimensions and tumor volume was calculated according to the formula V = (D × d^2^)/2, where D and d are the major and minor perpendicular tumor diameters, respectively^47^. Twenty four hours after the first treatment or at the end of the experimental procedures tumors were harvested, embedded in OCT-compound (Bio-Optica) and immediately frozen in liquid nitrogen for immunohistochemical analysis.

### Statistical analysis

Unless indicated differently, the results are presented as mean ± SEM. The differences between different treatments were analyzed, using the two-sided Student’s t test. P values lower than 0.05 were considered significant.

## Additional Information

**How to cite this article**: Porcù, E. *et al*. The Novel Antitubulin Agent TR-764 Strongly Reduces Tumor Vasculature and Inhibits HIF-1α Activation. *Sci. Rep.* **6**, 27886; doi: 10.1038/srep27886 (2016).

## Supplementary Material

Supplementary Information
