# Supplementary material for: The Novel Antitubulin Agent TR-764 Strongly Reduces Tumor Vasculature and Inhibits HIF-1α Activation
Source: Sci Rep. 2016 Jun 13;6:27886. doi: 10.1038/srep27886 (PMC4904223; doi:10.1038/srep27886)
Supplement: Supplementary Information [file srep27886-s1.pdf]

THE NOVEL ANTITUBULIN AGENT TR-764 STRONGLY REDUCES TUMOR VASCULATURE AND INHIBITS HIF-1α ACTIVATION

Elena Porcù,[1*] Luca Persano,[1] Roberto Ronca,[2] Stefania Mitola,[2] Roberta Bortolozzi,[1] Romeo Romagnoli,[3] Paola Oliva,[3] Giuseppe Basso,[1] Giampietro Viola[1*]

[1]*Dipartimento di Salute della Donna e del Bambino, Laboratorio di Oncoematologia pediatrica, Università di Padova, 35128 Padova, Italy.*

[2]*Dipartimento di Medicina molecolare e traslazionale Unità di oncologia sperimentale ed immunologia. Università di Brescia, 25123 Brescia, Italy.*

[3]*Dipartimento di Scienze Chimiche e Farmaceutiche, Università di Ferrara, 44121 Ferrara, Italy.*

[*]Correspondence to

Dr. Giampietro Viola

E-mail: giampietro.viola.1@unipd.it

and to

Dr. Elena Porcù

E-mail: elena.porcu@gmail.com

Dipartimento di Salute della Donna e del Bambino, Laboratorio di Oncoematologia pediatrica, Università di Padova, 35128 Padova, Italy.

Supplementary Data

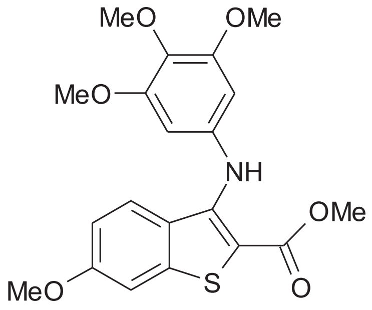

Supplementary Figure S1. Chemical structure of compound TR-764.

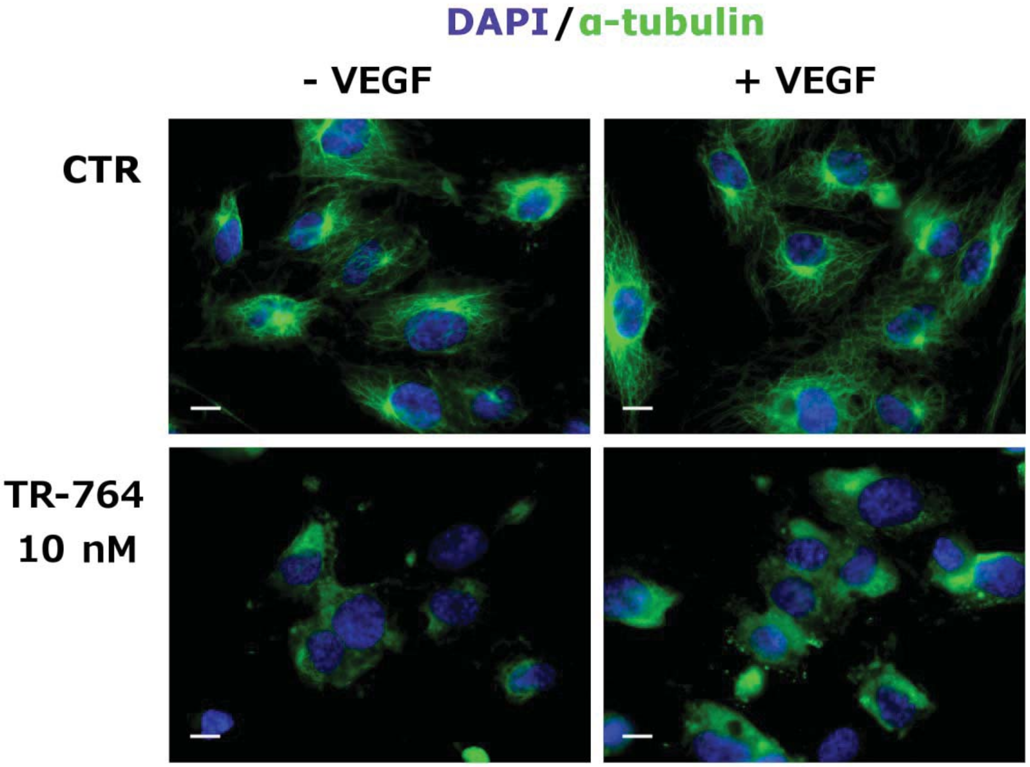

Supplementary Figure S2. TR-764 disrupts α-tubulin filaments. Immunofluorescence images of HUVECs treated with TR-764 10 nM for 6 h in presence and absence of VEGF. Cells were fixed and stained with primary antibodies anti- α-tubulin (green) and DAPI (blue) was used to visualize cell nuclei (60x magnification, scale bar = 10 μm).

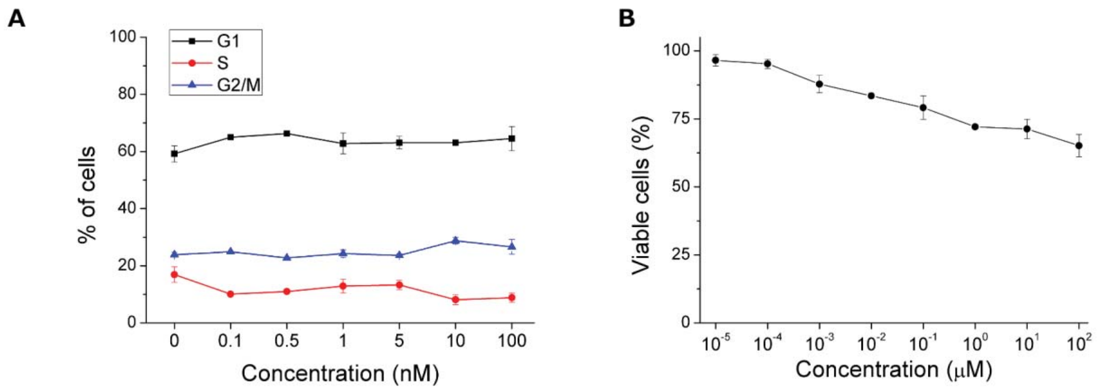

Supplementary Figure S3. TR-764 is not toxic for HUVEC cells. Cell cycle was analyzed after 24 h of treatment with TR-764 at the indicated concentrations (**A**). MTT test was performed after 72 h of treatment with TR-764 to evaluate cell proliferation (**B**).

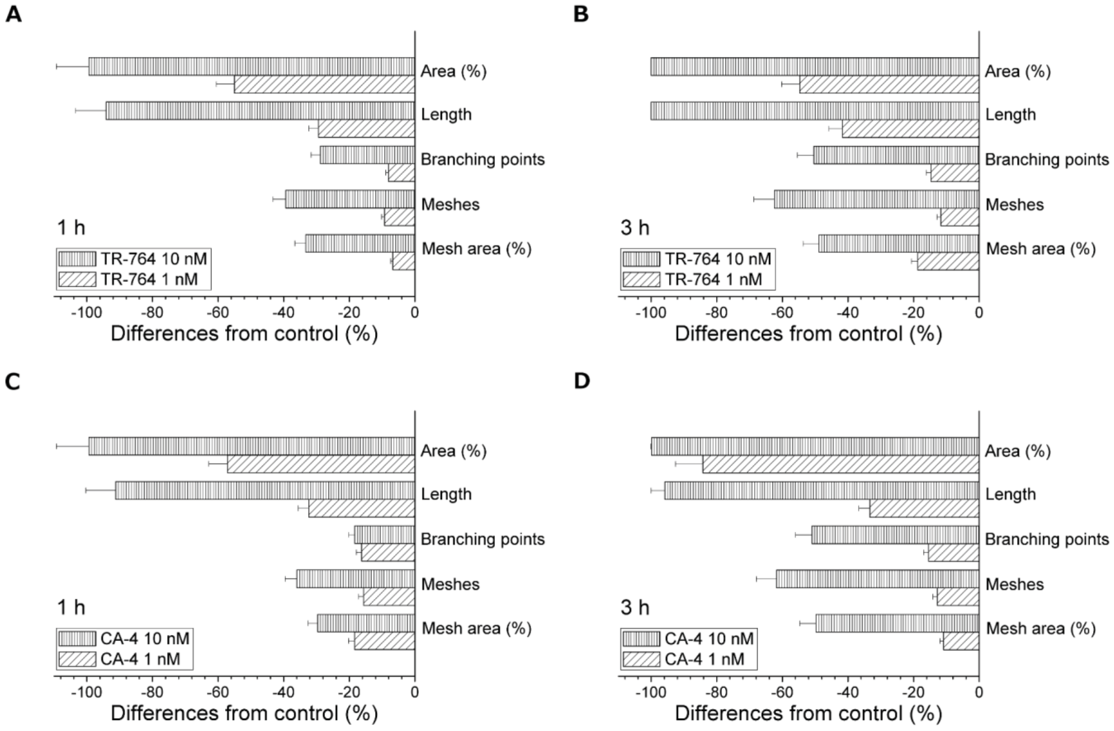

Supplementary Figure S4. Quantitative analysis of treatment with TR-764 for 1 h (**A**) or 3 h (**B**), and CA-4 for 1 h (**C**) or 3 h (**D**), on dimensional and topological parameters of tubule networks, formed by HUVECs seeded on Matrigel, in hypoxic conditions. Data were represented as mean ± SEM of three independent experiments.
